# Supplementary material for: PhpCNF-Y transcription factor infiltrates heterochromatin to generate cryptic intron-containing transcripts crucial for small RNA production
Source: Nat Commun. 2025 Jan 2;16:268. doi: 10.1038/s41467-024-55736-3 (PMC11696164; doi:10.1038/s41467-024-55736-3)
Supplement: Supplementary file 1 — Supplementary Information [file 41467_2024_55736_MOESM1_ESM.pdf]

Supplementary Information

PhpC^{NF-Y} transcription factor infiltrates heterochromatin to generate cryptic intron-containing transcripts crucial for small RNA production

Manjit Kumar Srivastav[1], H. Diego Folco[1], Patroula Nathanailidou[1], Anupa T Anil[1], Drisya Vijayakumari[1], Shweta Jain[1], Jothy Dhakshnamoorthy[1], Maura O'Neill[2], Thorkell Andresson[2], David Wheeler[1], and Shiv I.S. Grewal[1]*

Supplementary figures 1-8

*Corresponding author
Email: grewals@mail.nih.gov

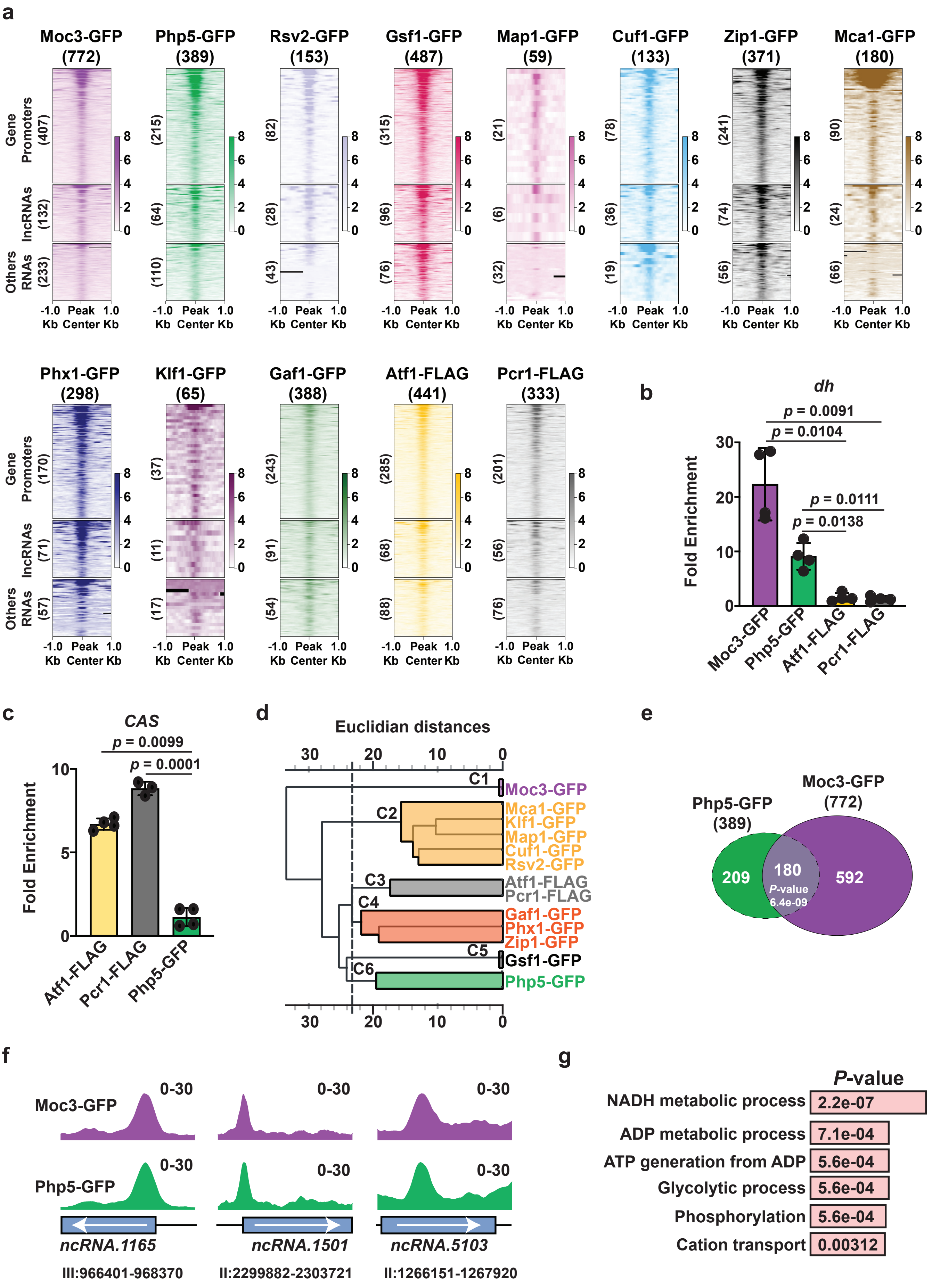
a
Moc3-GFP (772)
Php5-GFP (389)
Rsv2-GFP (153)
Gsf1-GFP (487)
Map1-GFP (59)
Cuf1-GFP (133)
Zip1-GFP (371)
Mca1-GFP (180)
Gene Promoters
lncRNAs
Others RNAs
(407) (132) (233)
(215) (64) (110)
(82) (28) (43)
(315) (96) (76)
(21) (6) (32)
(78) (36) (19)
(241) (74) (56)
(90) (24) (66)
-1.0 Kb Peak Center 1.0 Kb
Phx1-GFP (298)
Klf1-GFP (65)
Gaf1-GFP (388)
Atf1-FLAG (441)
Pcr1-FLAG (333)
(170) (71) (57)
(37) (11) (17)
(243) (91) (54)
(285) (68) (88)
(201) (56) (76)
b
dh
p = 0.0091
p = 0.0104
p = 0.0111
p = 0.0138
Fold Enrichment
Moc3-GFP
Php5-GFP
Atf1-FLAG
Pcr1-FLAG
c
CAS
p = 0.0099
p = 0.0001
Fold Enrichment
Atf1-FLAG
Pcr1-FLAG
Php5-GFP
d
Euclidian distances
C1 Moc3-GFP
C2 Mca1-GFP Klf1-GFP Map1-GFP Cuf1-GFP Rsv2-GFP
C3 Atf1-FLAG Pcr1-FLAG
C4 Gaf1-GFP Phx1-GFP Zip1-GFP
C5 Gsf1-GFP
C6 Php5-GFP
e
Php5-GFP (389)
Moc3-GFP (772)
209
180
P-value 6.4e-09
592
f
Moc3-GFP
Php5-GFP
0-30
ncRNA.1165
ncRNA.1501
ncRNA.5103
III:966401-968370
II:2299882-2303721
II:1266151-1267920
g
P-value
NADH metabolic process 2.2e-07
ADP metabolic process 7.1e-04
ATP generation from ADP 5.6e-04
Glycolytic process 5.6e-04
Phosphorylation 5.6e-04
Cation transport 0.00312

Supplementary Fig. 1 | Genome-wide distribution of TFs. (a) Peak-center heat map generated based on the binding events of the specified TFs at gene promoters, long non-coding RNAs (lncRNAs), and other non-coding RNAs (*tRNA*, *rRNAs*, *snoRNAs*). **(b, c)** ChIP-qPCR analysis of GFP-tagged TFs at the *dh* **(b)** and *CAS* **(c)** sites in wild-type strains. Data from at least 3 independent biological experiments are displayed as mean ± SD of the relative fold enrichment compared to the *leu1* control locus and normalized to the untagged strain. **(d)** Hierarchical cluster analysis of the genome-wide binding patterns of TFs. **(e)** Euler diagram displaying the number of loci bound by Php5 and Moc3. **(f)** Moc3-GFP and Php5-GFP enrichments are shown at specific lncRNA loci related to growth and stress. Genomic coordinates are shown below. **(g)** The most-enriched Gene Ontology (GO) biological process terms are shown among the 180 genes bound by Php5 and Moc3. Source data are provided as a Source Data file.

a

Biological replicate 1

	Protein	% Coverage Php5-GFP	% Coverage No tag Control
PhpC	Php5	52	0
PhpC	Php3	45	0
PhpC	Php2	4	0
	Moc3	23	0
	Atf1	31	0
	Pcr1	17	0
Rsc Complex	Rsc4	16	0
Rsc Complex	Rsc58	23	0
Rsc Complex	Rsc1	3	0
Rsc Complex	Rsc9	5	0
Rsc Complex	Snf21	17	0
Ino80 Complex	Ies6	37	0
Ino80 Complex	Ies4	19	0
Ino80 Complex	Iec5	43	0
Ino80 Complex	Iec3	34	0
Ino80 Complex	Ies2	4	0
Ino80 Complex	Ino80	11	0
Ino80 Complex	Iec1	18	0

Biological replicate 2

	Protein	% Coverage Php5-GFP	% Coverage No tag Control
Rsc Complex	Rsc4	44	0
Rsc Complex	Rsc58	38	0
Rsc Complex	Rsc1	21	0
Rsc Complex	Rsc9	13	0
Rsc Complex	Snf21	6	0
Ino80 Complex	Ies6	46	0
Ino80 Complex	Ies4	38	0
Ino80 Complex	Iec5	38	0
Ino80 Complex	Iec3	36	0
Ino80 Complex	Ies2	8	0
Ino80 Complex	Ino80	5	0
Ino80 Complex	Iec1	3	0

b

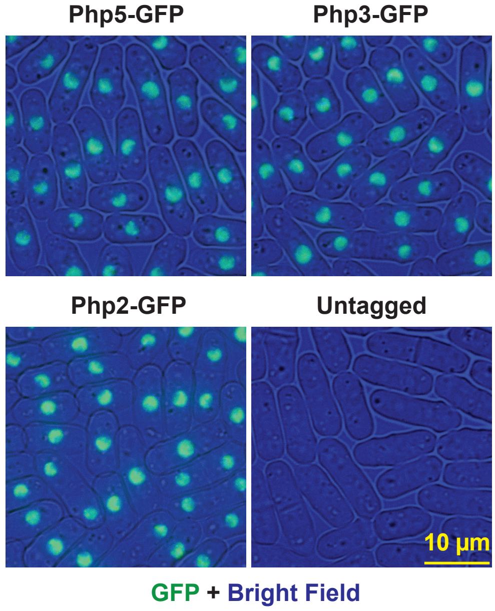

c

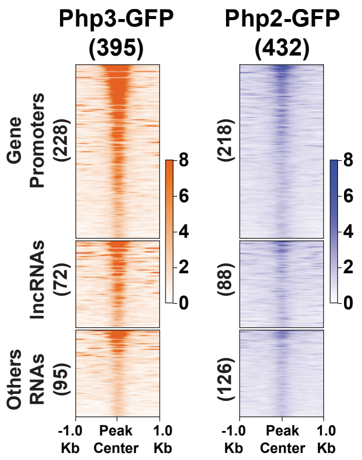

d

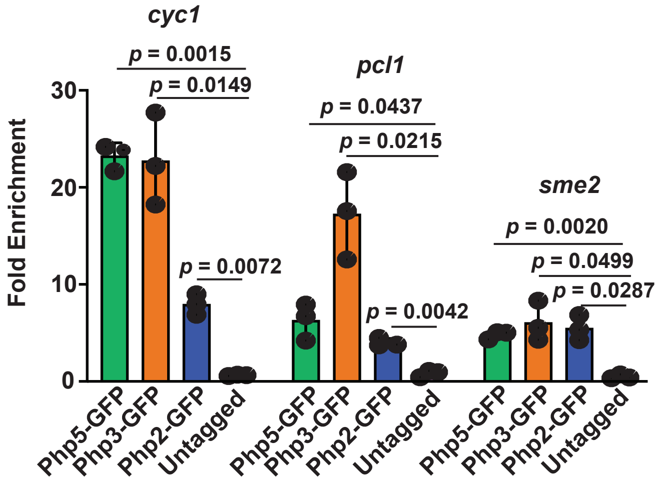

e

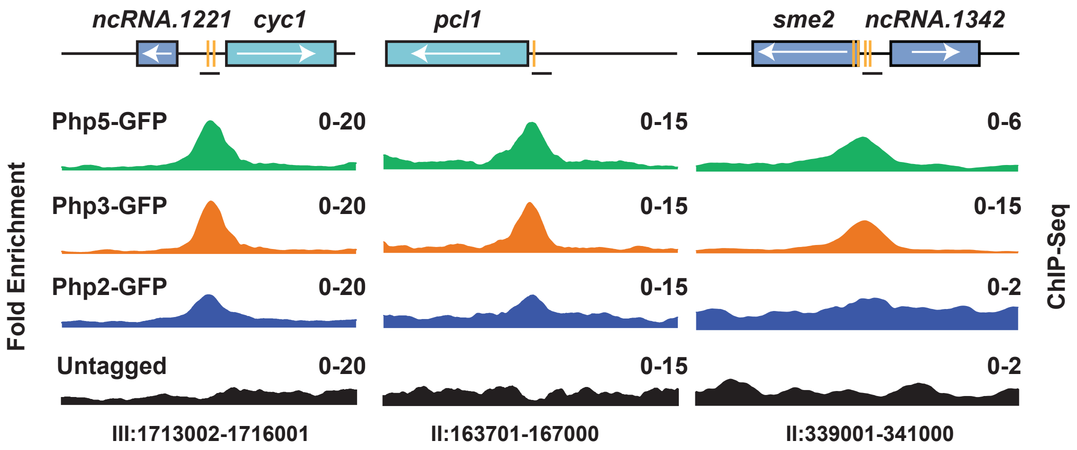

Supplementary Fig. 2 | Genome-wide distribution of PhpC subunits. (a) Immunopurified fractions, prepared from a strain expressing Php5-GFP and an untagged strain, were subjected to mass spectrometry. The total peptide coverage (%) for the indicated proteins is displayed. Data from two independent biological replicates are shown. See also Fig. 2b. **(b)** Representative live-cell images of strains expressing the indicated GFP-tagged PhpC subunits. **(c)** Peak-center heat maps generated based on the binding events of Php3 and Php2 at gene promoters, lncRNAs, and other non-coding RNAs (*tRNA*, *rRNAs*, *snoRNAs*). **(d)** ChIP-qPCR analysis of GFP-tagged PhpC subunits at the indicated loci. Data from 3 independent biological experiments are presented as the mean ± SD of the relative fold enrichment compared to the *leu1* control locus. **(e)** ChIP-seq analysis of the enrichment of PhpC subunits. Vertical yellow lines in the schematic indicate *CCAAT* boxes. The black horizontal lines in the schematic indicate the annealing sites of the primers used in panel **d.** Genomic coordinates are shown below. Source data are provided as a Source Data file.

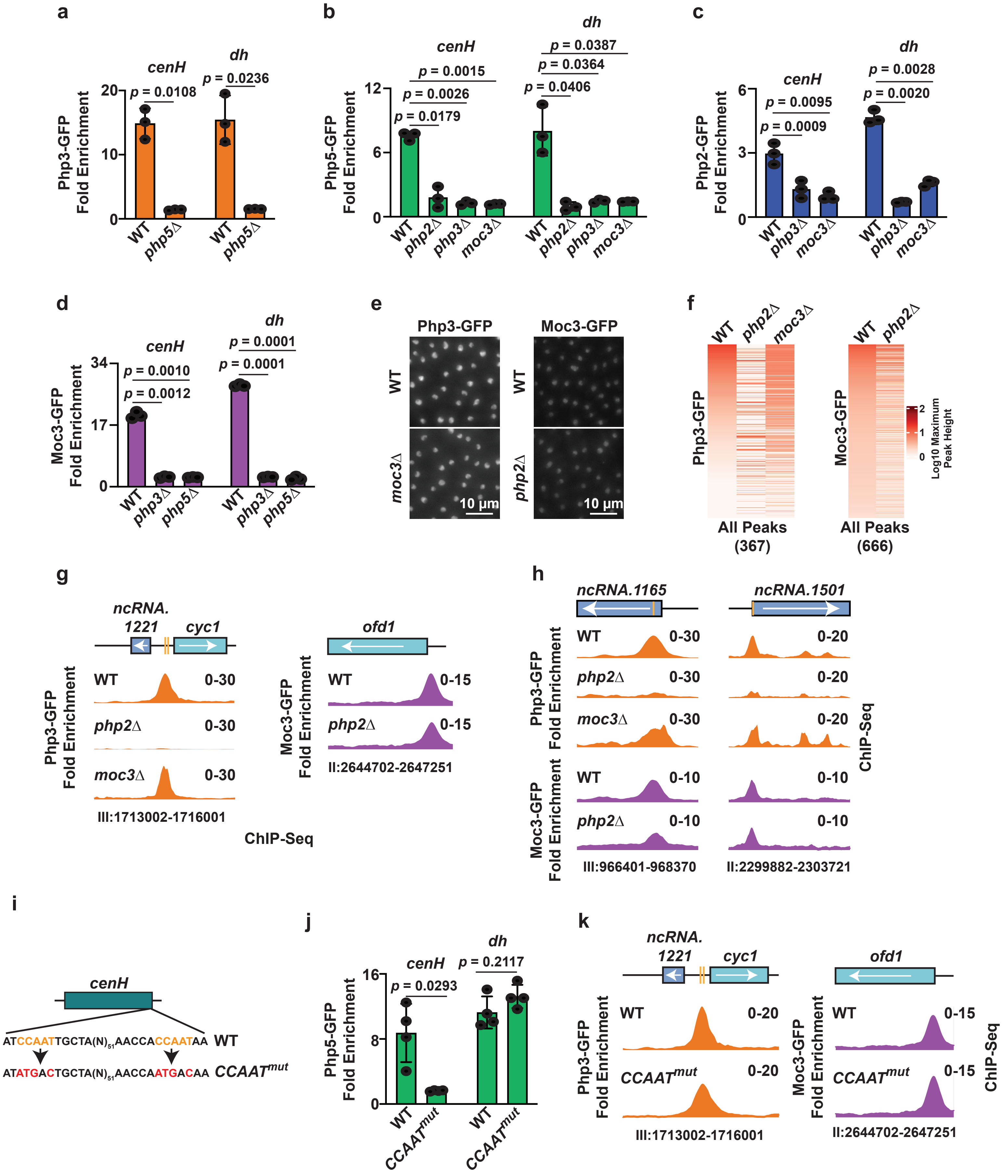
a
Php3-GFP Fold Enrichment
cenH
dh
p = 0.0108
p = 0.0236
WT
php5Δ
b
Php5-GFP Fold Enrichment
p = 0.0179
p = 0.0026
p = 0.0015
p = 0.0406
p = 0.0364
p = 0.0387
php2Δ
php3Δ
moc3Δ
c
Php2-GFP Fold Enrichment
p = 0.0009
p = 0.0095
p = 0.0020
p = 0.0028
d
Moc3-GFP Fold Enrichment
p = 0.0012
p = 0.0010
p = 0.0001
p = 0.0001
e
Php3-GFP
Moc3-GFP
10 μm
f
Log10 Maximum Peak Height
All Peaks (367)
All Peaks (666)
g
ncRNA. 1221
cyc1
ofd1
III:1713002-1716001
II:2644702-2647251
ChIP-Seq
h
ncRNA.1165
ncRNA.1501
III:966401-968370
II:2299882-2303721
i
ATCCAATTGCTA(N)51AACCACCAATAA WT
ATATGACTGCTA(N)51AACCAATGACAA CCAATmut
j
p = 0.0293
p = 0.2117
CCAATmut
k

Supplementary Fig. 3 | Binding of PhpC and Moc3 at heterochromatic regions is interdependent. (a-d) ChIP-qPCR analysis was performed at *cenH* and *dh* in strains expressing GFP-tagged Php3 **(a)**, Php5 **(b)**, Php2 **(c)** and Moc3 **(d)**. Note that the WT ChIP-qPCR data for Moc3-GFP is the same as in Fig. 3c**,** as the experiments were performed simultaneously. **(e)** Representative live-cell images of strains expressing GFP-tagged Php3 or Moc3. **(f)** Heat maps showing the relative ChIP-seq enrichments for Php3-GFP (left) and Moc3-GFP (right) in the indicated strains. **(g)** ChIP-seq enrichments of Php3-GFP and Moc3-GFP in the indicated strains at *cyc1* and *ofd1* loci. WT Php3-GFP ChIP-seq data is replotted from Supplementary Fig. 2e. **(h)** Php3-GFP and Moc3-GFP ChIP-seq enrichments at select loci. WT Moc3-GFP ChIP-seq data is replotted from Supplementary Fig. 1f. **(i)** Schematic depicting the CRISPR-Cas9 editing of two *CCAAT* boxes at *cenH*. **(j)** ChIP-qPCR analysis of Php5-GFP was performed at *cenH* and *dh* in wild-type and *CCAAT*^{mut} strains. **(k)** ChIP-seq analysis of Php3-GFP enrichment at a PhpC target locus (left) and Moc3-GFP enrichment at a select locus (right) in the indicated strains. WT ChIP-seq data is replotted from Supplementary Fig. 3g. Genomic coordinates are shown below **(g, h, k).** For a-d and j, data from at least 3 independent biological experiments are presented as the mean ± SD of the relative fold enrichment compared to the *leu1* control locus. Source data are provided as a Source Data file.

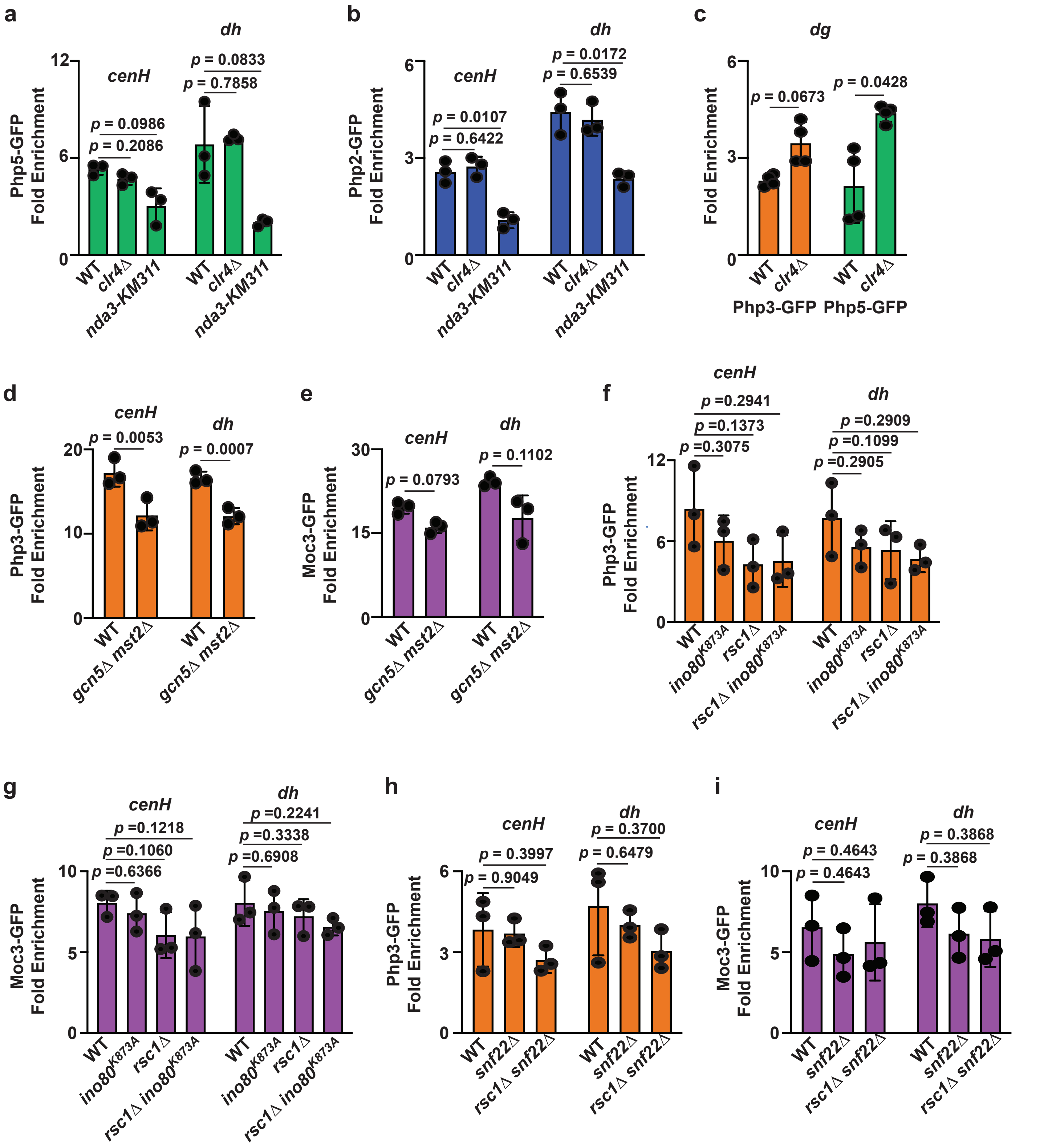

Supplementary Fig. 4 | PhpC and Moc3 localization to heterochromatin is maintained in cells lacking HATs and chromatin remodelers. (a, b) ChIP-qPCR analysis of Php5-GFP **(a)** or Php2-GFP **(b)** enrichment at *cenH* and *dh* in the indicated strains. **(c)** ChIP-qPCR analysis of GFP-tagged Php3 or Php5 at *dg* in the indicated strains. The location where the primer binds is indicated in the schematic in Fig. 4b by black horizontal lines. **(d-i)** ChIP-qPCR analysis of GFP-tagged Php3 **(d, f, h)** or Moc3 **(e, g, i)** enrichment at *cenH* and *dh* in the indicated strains. Data from at least 3 independent biological experiments are presented as the mean ± SD. Source data are provided as a Source Data file.

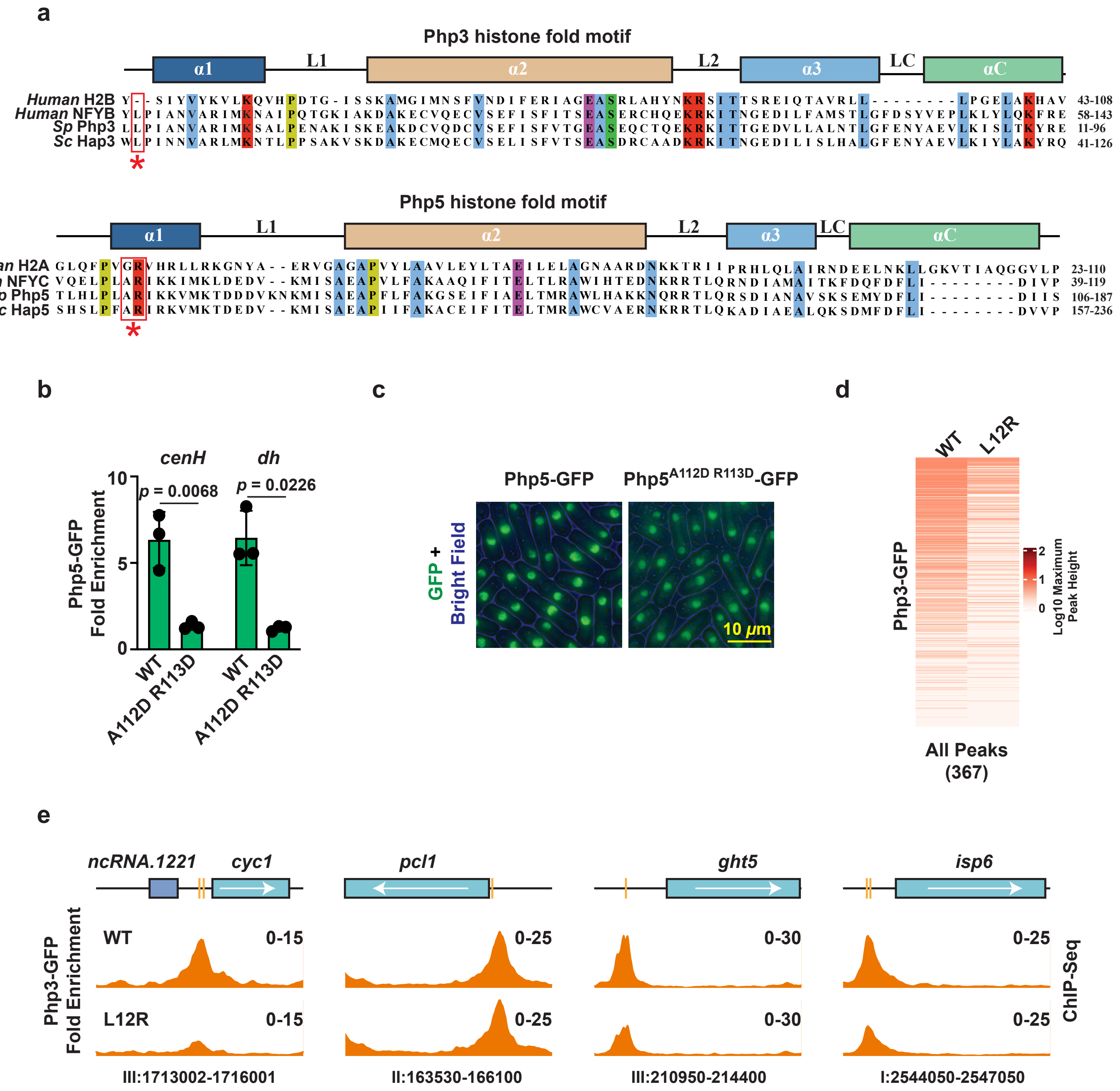

Supplementary Fig. 5 | Mutation of conserved residues in the histone fold domains of PhpC abolishes its localization to heterochromatin. (a) Alignments of the human H2B *(H2BC3)* and H2A *(H2AC1)* histone fold domains with human NF-Y subunits NF-YB and NF-YC, *S. pombe* PhpC subunits Php3 and Php5 and *Saccharomyces cerevisiae* Hap3 and Hap5. The corresponding amino acid numbers are shown on the right. **(b)** ChIP-qPCR analysis of GFP-tagged Php5 enrichment at *cenH* and *dh* in the indicated strains. Data from 3 independent biological experiments are presented as the mean ± SD. **(c)** Representative live-cell images of strains expressing GFP-tagged Php5 wild type and mutant proteins. **(d)** Heat maps showing the relative ChIP-seq enrichments for GFP-tagged Php3 in the indicated strains. **(e)** ChIP-seq enrichments of Php3-GFP at selected loci in the indicated strains. Genomic coordinates are shown below. Source data are provided as a Source Data file.

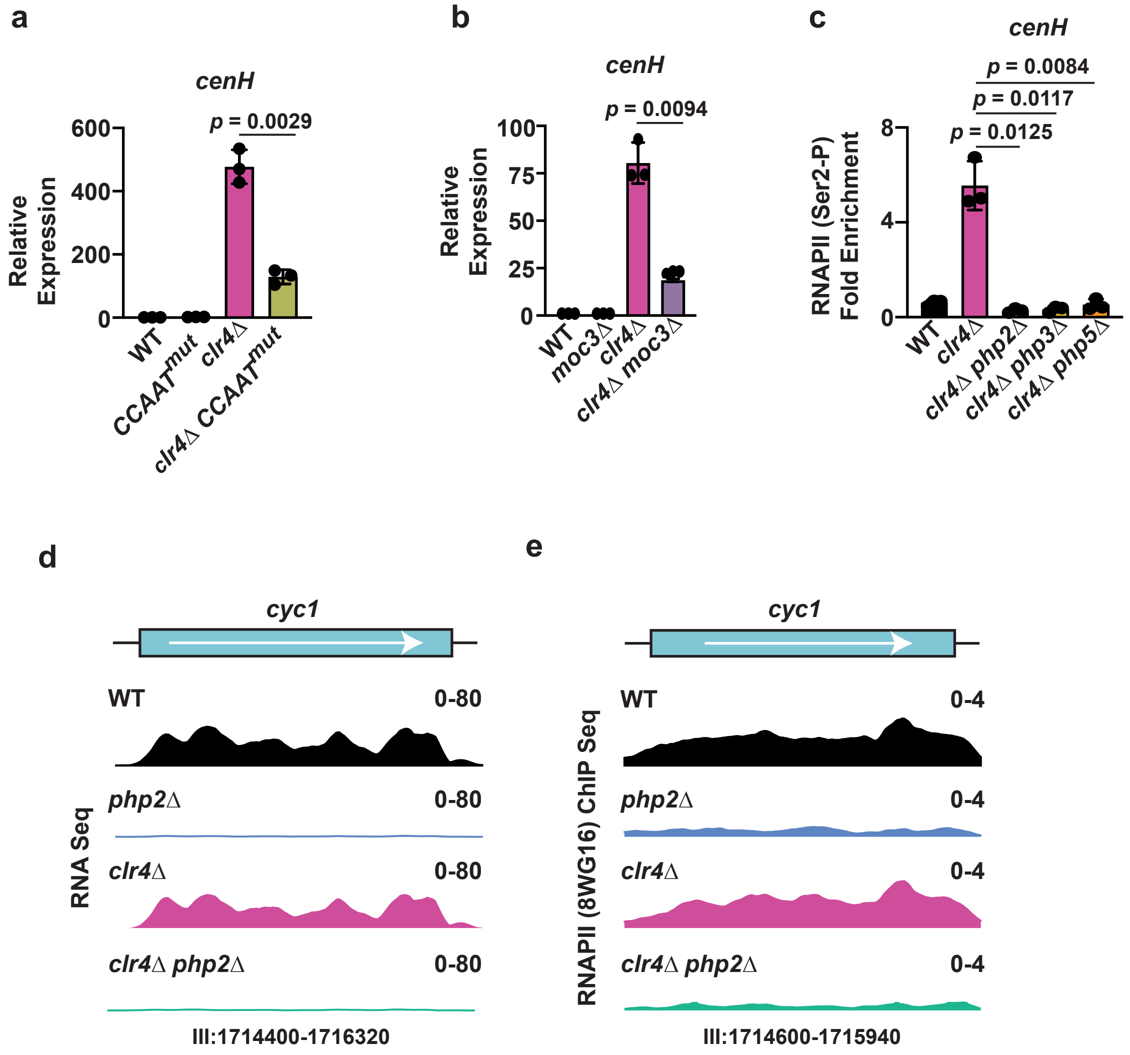

Supplementary Fig. 6 | PhpC and Moc3 promote transcription and RNAPII enrichment at *cenH*. (a, b) RT-qPCR analysis of *cenH* transcripts in the indicated strains. The *leu1* locus was used as a control. **(c)** ChIP-qPCR analysis of RNAPII occupancy at *cenH* in the indicated strains using anti-Ser2-P antibodies. Data is presented as relative fold enrichment (ChIP) compared to the *tRNA* control locus. **(d, e)** RNA-seq expression profile **(d)** and RNAPII ChIP-seq enrichment **(e)** at *cyc1* in the indicated strains. Genomic coordinates are shown below. Data from 3 independent biological experiments are presented as the mean ± SD **(a-c).** Source data are provided as a Source Data file.

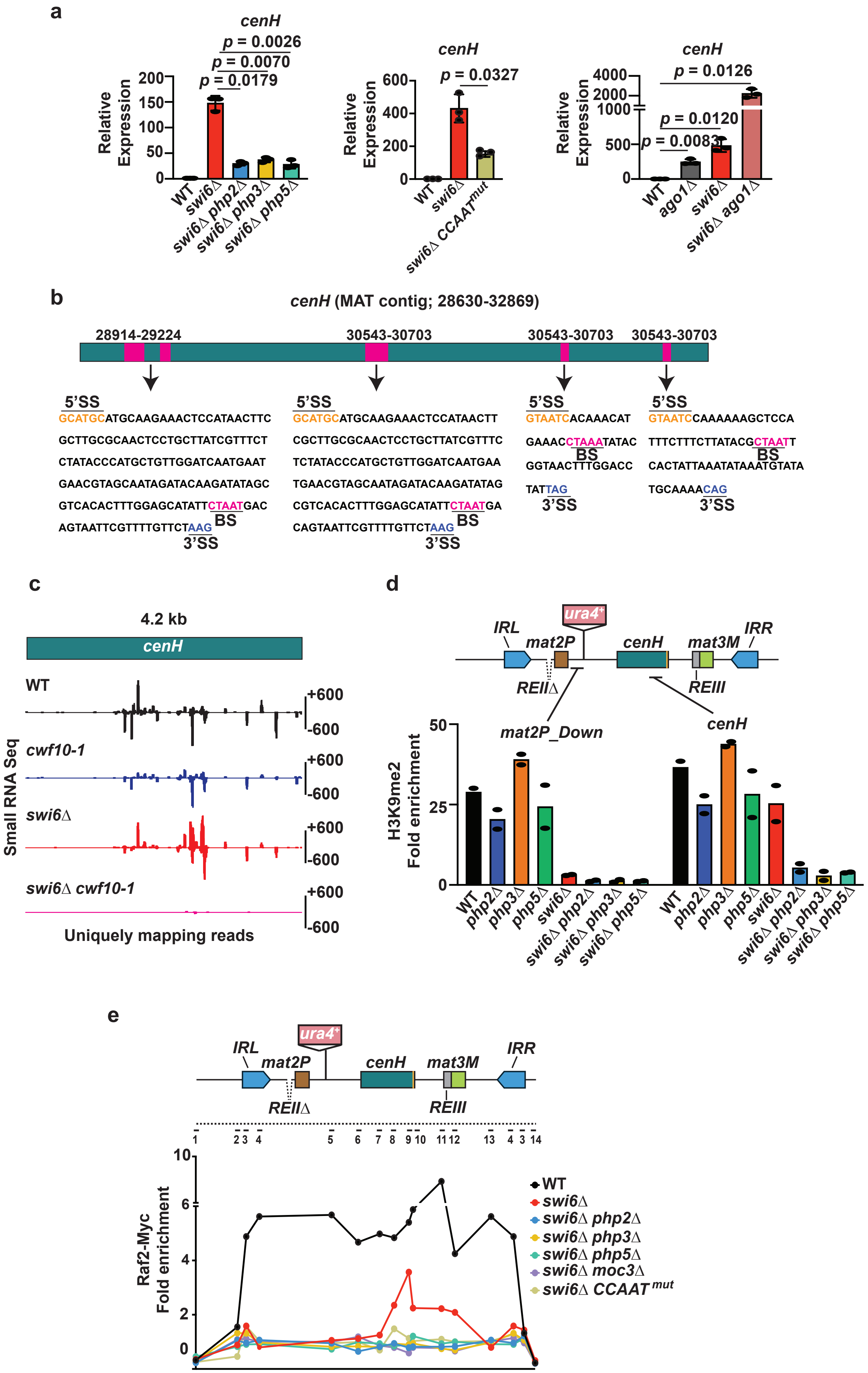
a
cenH
p = 0.0026
p = 0.0070
p = 0.0179
Relative Expression
200
150
100
50
0
WT
swi6Δ
swi6Δ php2Δ
swi6Δ php3Δ
swi6Δ php5Δ
cenH
p = 0.0327
600
400
200
0
WT
swi6Δ
swi6Δ CCAAT^mut
cenH
p = 0.0126
4000
2000
1000
500
0
p = 0.0120
p = 0.0083
WT
ago1Δ
swi6Δ
swi6Δ ago1Δ
b
cenH (MAT contig; 28630-32869)
28914-29224
30543-30703
30543-30703
30543-30703
5'SS
GCATGCATGCAAGAAACTCCATAACTTC
GCTTGCGCAACTCCTGCTTATCGTTTCT
CTATACCCATGCTGTTGGATCAATGAAT
GAACGTAGCAATAGATACAAGATATAGC
GTCACACTTTGGAGCATATTCTAATGAC
AGTAATTCGTTTTGTTCTAAG
BS
3'SS
5'SS
GCATGCATGCAAGAAACTCCATAACTT
CGCTTGCGCAACTCCTGCTTATCGTTTC
TCTATACCCATGCTGTTGGATCAATGAA
TGAACGTAGCAATAGATACAAGATATAG
CGTCACACTTTGGAGCATATTCTAATGA
CAGTAATTCGTTTTGTTCTAAG
BS
3'SS
5'SS
GTAATCACAAACAT
GAAACCTAAATATAC
BS
GGTAACTTTGGACC
TATTAG
3'SS
5'SS
GTAATCCAAAAAAGCTCCA
TTTCTTTCTTATACGCTAATT
BS
CACTATTAAATATAAATGTATA
TGCAAAACAG
3'SS
c
4.2 kb
cenH
Small RNA Seq
WT
cwf10-1
swi6Δ
swi6Δ cwf10-1
+600
-600
Uniquely mapping reads
d
ura4+
IRL
mat2P
cenH
mat3M
IRR
REIIΔ
REIII
mat2P_Down
cenH
H3K9me2 Fold enrichment
50
25
0
WT
php2Δ
php3Δ
php5Δ
swi6Δ
swi6Δ php2Δ
swi6Δ php3Δ
swi6Δ php5Δ
e
ura4+
IRL
mat2P
cenH
mat3M
IRR
REIIΔ
REIII
1 2 3 4 5 6 7 8 9 10 11 12 13 4 3 14
Raf2-Myc Fold enrichment
10
6
4
2
0
WT
swi6Δ
swi6Δ php2Δ
swi6Δ php3Δ
swi6Δ php5Δ
swi6Δ moc3Δ
swi6Δ CCAAT^mut

Supplementary Fig. 7 | The *cenH* bottom strand harbors cryptic introns. (a) RT-qPCR analysis of *cenH* transcripts in the indicated strains is shown as the relative fold expression compared to the control *leu1* locus. Data from 3 independent biological experiments are presented as the mean ± SD. (**b)** Schematic depicting the location of cryptic introns in the bottom strand of *cenH* transcripts. The DNA sequence of each cryptic intron is provided, and the locations of 5' and 3' splice and branch sites are indicated. **(c)** siRNA-seq profiles at the *cenH* region in the indicated strains. Note that the reads were uniquely mapped to *cenH*. **(d)** ChIP-qPCR analysis of H3K9me2 enrichment at two *mat* locations (see schematic) in the indicated strains. **(e)** ChIP-qPCR analysis of Raf2-myc at the silent *mat* region in the indicated strains. Data is presented as the mean of 2 independent biological experiments **(d-e).** Numbered lines in the schematic indicate the location of primers. Source data are provided as a Source Data file.

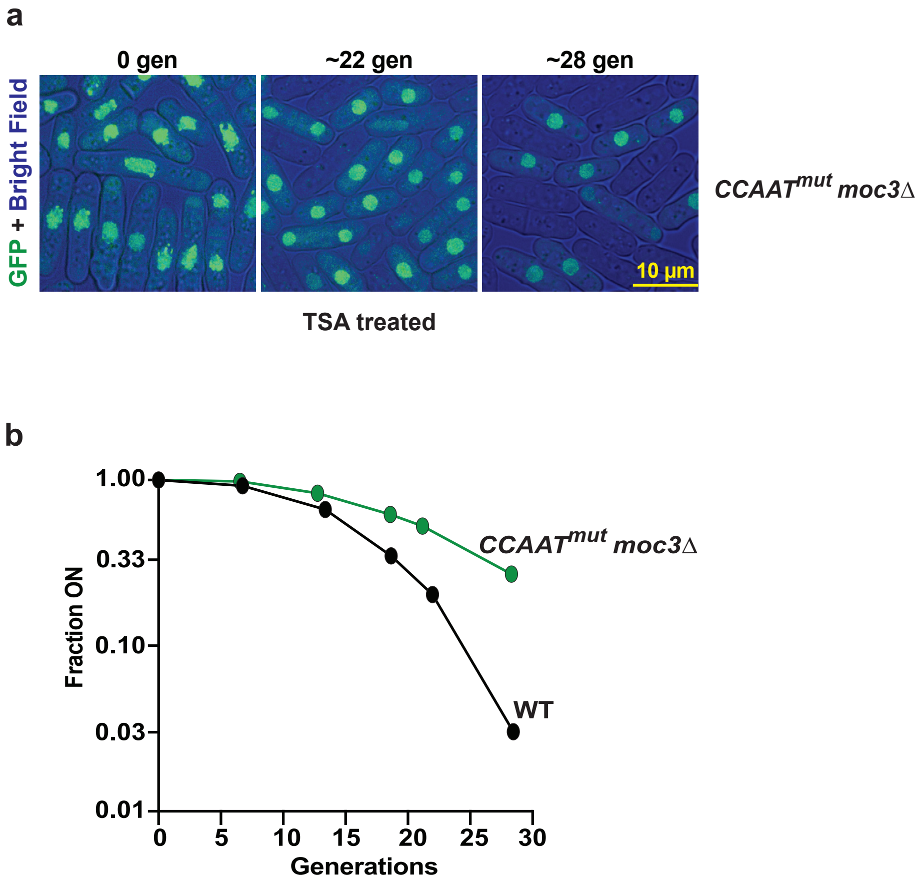

Supplementary Fig. 8 | Moc3 contributes to *de novo* heterochromatin establishment. **(a)** Live-cell imaging of a *CCAAT*^{mut} *moc3Δ* strain containing the *mat2P::GFP* reporter at the indicated time points (generations) post-TSA treatment and washout. **(b)** The fraction of cells in the "ON" state in WT and *CCAAT*^{mut} *moc3Δ* strains at the indicated time points are quantified. N= 207-646 cells for each data point. WT data is replotted from Fig.7d, as the experiments were performed simultaneously. Source data are provided as a Source Data file.
