# Supplementary material for: PhpCNF-Y transcription factor infiltrates heterochromatin to generate cryptic intron-containing transcripts crucial for small RNA production
Source: Nat Commun. 2025 Jan 2;16:268. doi: 10.1038/s41467-024-55736-3 (PMC11696164; doi:10.1038/s41467-024-55736-3)
Supplement: Supplementary file 3 — Description of Additional Supplementary Files [file 41467_2024_55736_MOESM3_ESM.pdf]

**Description of Additional Supplementary Files**

File Name: **Supplementary Data 1** Description: List of loci showing TF enrichment.

File Name: **Supplementary Data 2** Description: List of loci showing either Php5, Moc3 or both Php5 and Moc3 enrichment.

File Name: **Supplementary Data 3** Description: Mapping of PhpC components across the genome.

File Name: **Supplementary Data 4** Description: Mapping of Php3 and Moc3 in the indicated mutant background.

File Name: **Supplementary Data 5** Description: List of strains and oligos used in this study.
