# Supplementary material for: PhpCNF-Y transcription factor infiltrates heterochromatin to generate cryptic intron-containing transcripts crucial for small RNA production
Source: Nat Commun. 2025 Jan 2;16:268. doi: 10.1038/s41467-024-55736-3 (PMC11696164; doi:10.1038/s41467-024-55736-3)
Supplement: Supplementary file 9 — Reporting Summary [file 41467_2024_55736_MOESM9_ESM.pdf]

nature portfolio

Corresponding author(s): Shiv Grewal

Last updated by author(s): Dec 6, 2024

Reporting Summary

Nature Portfolio wishes to improve the reproducibility of the work that we publish. This form provides structure for consistency and transparency in reporting. For further information on Nature Portfolio policies, see our Editorial Policies and the Editorial Policy Checklist.

Statistics

For all statistical analyses, confirm that the following items are present in the figure legend, table legend, main text, or Methods section.

n/a	Confirmed	
☐	☒	The exact sample size (*n*) for each experimental group/condition, given as a discrete number and unit of measurement
☐	☒	A statement on whether measurements were taken from distinct samples or whether the same sample was measured repeatedly
☐	☒	The statistical test(s) used AND whether they are one- or two-sided<br>*Only common tests should be described solely by name; describe more complex techniques in the Methods section.*
☒	☐	A description of all covariates tested
☒	☐	A description of any assumptions or corrections, such as tests of normality and adjustment for multiple comparisons
☐	☒	A full description of the statistical parameters including central tendency (e.g. means) or other basic estimates (e.g. regression coefficient) AND variation (e.g. standard deviation) or associated estimates of uncertainty (e.g. confidence intervals)
☐	☒	For null hypothesis testing, the test statistic (e.g. *F*, *t*, *r*) with confidence intervals, effect sizes, degrees of freedom and *P* value noted<br>*Give P values as exact values whenever suitable.*
☒	☐	For Bayesian analysis, information on the choice of priors and Markov chain Monte Carlo settings
☒	☐	For hierarchical and complex designs, identification of the appropriate level for tests and full reporting of outcomes
☒	☐	Estimates of effect sizes (e.g. Cohen's *d*, Pearson's *r*), indicating how they were calculated

Our web collection on statistics for biologists contains articles on many of the points above.

Software and code

Policy information about availability of computer code

Data collection	Microscopy imaging was performed using a Delta Vision Elite microscope (Leica). ChIP-seq data were obtained with the next-generation Illumina MiSeq sequencer. RNA-seq and Small RNA-seq data were collected using the Illumina NextSeq 500/550 sequencer. Western blot signals were acquired using the Supersignal West Pico PLUS Chemiluminescent substrate (Thermo Fisher) and Amersham Hyperfilm ECL, which was developed using a Konica SRX-101A.
Data analysis	Microscopy image processing, which includes deconvolution and maximum intensity projection, was performed using ImageJ Fiji and SoftWorx.<br>ChIP-seq and RNA-seq data were visualized using the Integrated Genome Browser (IGV, version 2.17.4), while small RNA-seq data were visualized in the Integrated Genome Browser (IGB). ChIP-seq reads were trimmed using fastp and aligned to the S. pombe V2 reference sequence with the BWA aligner. The MACS2 callpeaks function was utilized to produce ChIP enrichment Bedgraphs, followed by the bdgcmp function to compute fold enrichment over the input. The Integrative Genomics Viewer (version 2.17.4) was used to plot enrichments along the fission yeast chromosomes. For RNA-seq, reads were also trimmed with fastp and aligned to the S. pombe V2 reference sequence, first using the STAR aligner and then with Novoalign software.<br>Serial dilution plates were scanned using an Epson Perfection V700 Photo scanner and presented using Adobe Photoshop (version 22.4.2) and Illustrator (2024).<br>Statistical parameters, including the mean fold enrichment (MFE) and the standard error of the mean (SEM), were calculated and plotted using Prism for Mac (version 10, GraphPad Software).

For manuscripts utilizing custom algorithms or software that are central to the research but not yet described in published literature, software must be made available to editors and reviewers. We strongly encourage code deposition in a community repository (e.g. GitHub). See the Nature Portfolio guidelines for submitting code & software for further information.

Data

Policy information about availability of data

All manuscripts must include a data availability statement. This statement should provide the following information, where applicable:

- Accession codes, unique identifiers, or web links for publicly available datasets
- A description of any restrictions on data availability
- For clinical datasets or third party data, please ensure that the statement adheres to our policy

ChIP-seq, RNAseq and small RNA Seq data are deposited in GEO under GSE269096 accession number and are publicly available.
Go to https://www.ncbi.nlm.nih.gov/geo/query/acc.cgi?acc=GSE269096.
Mass spectrometry data is deposited in MassIVE under dataset identifiers number MSV000095108 and are publicly available.
Go to https://massive.ucsd.edu/ProteoSAFe/dataset.jsp?accession=MSV000095108
Source data are provided with this paper in the Source Data file.

Research involving human participants, their data, or biological material

Policy information about studies with human participants or human data. See also policy information about sex, gender (identity/presentation), and sexual orientation and race, ethnicity and racism.

Reporting on sex and gender	N/A
Reporting on race, ethnicity, or other socially relevant groupings	N/A
Population characteristics	N/A
Recruitment	N/A
Ethics oversight	N/A

Note that full information on the approval of the study protocol must also be provided in the manuscript.

Field-specific reporting

Please select the one below that is the best fit for your research. If you are not sure, read the appropriate sections before making your selection.

[x] Life sciences [ ] Behavioural & social sciences [ ] Ecological, evolutionary & environmental sciences

For a reference copy of the document with all sections, see nature.com/documents/nr-reporting-summary-flat.pdf

Life sciences study design

All studies must disclose on these points even when the disclosure is negative.

Sample size	Sample size selection was based on experience from prior studies: [Holla et al., 2020, Cell (PMID: 31883795); Cutter DiPiazza et al., 2021, PNAS (PMID: 34035174); Lee et al., 2013, Cell (PMID: 24210919); Vo et al., 2019, Cell Rep (PMID: 31269446)]
Data exclusions	No data were excluded from analyses.
Replication	For all experiments, at least two biological replicates were performed.
Randomization	The experiments were not randomized.
Blinding	The Investigators were not blinded to allocation during experiments and outcome assessment.

Reporting for specific materials, systems and methods

We require information from authors about some types of materials, experimental systems and methods used in many studies. Here, indicate whether each material, system or method listed is relevant to your study. If you are not sure if a list item applies to your research, read the appropriate section before selecting a response.

Materials & experimental systems

n/a	Involved in the study
☐	☒ Antibodies
☒	☐ Eukaryotic cell lines
☒	☐ Palaeontology and archaeology
☒	☐ Animals and other organisms
☒	☐ Clinical data
☒	☐ Dual use research of concern
☒	☐ Plants

Methods

n/a	Involved in the study
☐	☒ ChIP-seq
☒	☐ Flow cytometry
☒	☐ MRI-based neuroimaging

Antibodies

Antibodies used	ChIP experiments were performed by combining 1 ml of pre-cleared cell lysate with appropriate antibody:<br>2µg of anti-H3K9me3 (Abcam, Cat#ab8898),<br>2µg of anti-H3K9me2 (Abcam, Cat#ab115159),<br>2µg of anti-GFP (Abcam, Cat#ab290),<br>2µg of anti-RNAPII (8WG16, Santa Cruz Biotechnology, sc56767),<br>2µg of anti-myc (Santa Cruz Biotechnology, Cat#9E10),<br>50µl anti-FLAG M2 affinity gel (Sigma, Cat#A2220),<br>2µg anti-RNAPII phospho S2 (Abcam ab5095),<br>For Php5-GFP western blot; 1:1000 dilution anti-GFP antibody (Roche Cat#11814460001)<br>For Php5-GFP Immunoprecipitation and Mass Spectrometry; 25µl anti-GFP agarose beads (GFP-Trap Chromotek, Cat#AB_2631357).<br><br>Western blot experiments were performed with anti-GFP antibody (Roche Cat#11814460001) diluted to 1:1000 dilution. We use 1:5000 dilution of secondary anti-mouse IgG1 (PA1-86329) HRP conjugated antibodies (Thermofisher Scientific).
Validation	All antibodies used in this study are established, commercially available clones that have been validated by the manufacturer and scientific literature. Validation data can be accessed on the manufacturer's website.

Plants

Seed stocks	N/A
Novel plant genotypes	N/A
Authentication	N/A

ChIP-seq

Data deposition

☒ Confirm that both raw and final processed data have been deposited in a public database such as GEO.

☒ Confirm that you have deposited or provided access to graph files (e.g. BED files) for the called peaks.

Data access links<br>*May remain private before publication.*	ChIP-seq, RNAseq and small RNA Seq data are deposited in GEO under GSE269096 accession number and are publicly available. To review GEO accession GSE269096:<br>Go to https://www.ncbi.nlm.nih.gov/geo/query/acc.cgi?acc=GSE269096<br><br>Mass spectrometry data is deposited in MassIVE under dataset identifiers number MSV000095108 and are publicly available.<br>Go to https://massive.ucsd.edu/ProteoSAFe/dataset.jsp?accession=MSV000095108
Files in database submission	Please see above
Genome browser session<br>(e.g. UCSC)	N/A

Methodology

Replicates	ChIP-Seq; Two biological replicates were performed for untagged, Php5-GFP, Php3-GFP, Php2-GFP, Moc3-GFP, Atf1-FLAG, Pcr1-FLAG and Gaf1-GFP in wild-type, and two biological replicates were performed for Php3-GFP, Moc3-GFP in different mutants background, others are performed one biological replicates.<br>IP_MS;Two biological replicates were performed for all samples.<br>RNA Seq; Two biological replicates were performed for all samples.<br>Small RNA-Seq; Two biological replicates were performed for all samples.
Sequencing depth	ChIP Seq libraries were sequenced to ~1-3million reads.<br>RNA Seq libraries were sequenced to ~20-50million reads.<br>Small RNA Seq libraries were sequenced to ~40million reads.
Antibodies	2µg of anti-H3K9me3 (Abcam, Cat#ab8898),<br>2µg of anti-GFP (Abcam, Cat#ab290),<br>2µg of anti-RNAPII (8WG16, Santa Cruz Biotechnology, sc56767),<br>50µl anti-FLAG M2 affinity gel (Sigma, Cat#A2220).
Peak calling parameters	--broad, --nomodel, --extsize 147 -g 12.57e6
Data quality	Reads were quality-trimmed using fastp. QC reports are generated by fastp.
Software	fastp (read trimming), BWA (alignment of trimmed reads), MACS2 (generation of fold enrichment bedgraphs), STAR aligner, Novoalign
